# High frequency of *kdr* L1014F is associated with pyrethroid resistance *in Anopheles coluzzii* in Sudan savannah of northern Nigeria

**DOI:** 10.1186/1471-2334-14-441

**Published:** 2014-08-15

**Authors:** Sulaiman S Ibrahim, Yayo A Manu, Zainab Tukur, Helen Irving, Charles S Wondji

**Affiliations:** Bayero University, P.M.B. 3011, Kano, Nigeria; Vector Biology Department, Liverpool School of Tropical Medicine, Pembroke Place, L3 5QA Liverpool, UK

**Keywords:** *An. coluzzii*, *An. arabiensis*, Nigeria, Savannah, Target-site mutations, *kdr*, Vector control

## Abstract

**Background:**

Malaria burden is high in Nigeria, yet information on the major mosquito vectors is lacking especially in the Sudan savannah region of the country. In order to facilitate the design of future insecticide-based control interventions in the region, this study has established the resistance profile of *An. gambiae s.l.* populations in two northern Nigeria locations and assessed the contribution of target site resistance mutations.

**Methods:**

Larval collection was conducted in two localities in Sudan savannah (Bunkure and Auyo) of northern Nigeria between 2009 and 2011, from which resulting adult, female mosquitoes were used for insecticides bioassays with deltamethrin, lambda-cyhalothrin, DDT and malathion. The mosquitoes were identified to species level and molecular forms and then genotyped for the presence of *L1014F-kdr*, *L1014S-kdr* and *ace-1*^*R*^ mutations.

**Results:**

WHO bioassays revealed that *An. gambiae s.l.* from both localities were highly resistant to lambda-cyhalothrin and DDT, but only moderately resistant to deltamethrin. Full susceptibility was observed to malathion. *An. gambiae*, M form (now *An. coluzzii*), was predominant over *An. arabiensis* in Auyo and was more resistant to lambda-cyhalothrin than *An. arabiensis*. No ‘S’ form (*An. gambiae s.s*.) was detected. A high frequency of 1014 F mutation (80.1%) was found in *An. coluzzii* in contrast to *An. arabiensis* (13.5%). The presence of the 1014 F *kdr* allele was significantly associated with resistance to lambda-cyhalothrin in *An. coluzzii* (OR = 9.85; P < 0.001) but not in *An. arabiensis*. The L1014S-*kdr* mutation was detected in a single *An. arabiensis* mosquito while no *ace-1*^*R*^ mutation was found in any of the mosquitoes analysed.

**Conclusions:**

The predominance of *An. coluzzii* and its resistance profile to main insecticides described in this study can guide the implementation of appropriate vector control interventions in this region of Nigeria where such information was previously lacking.

**Electronic supplementary material:**

The online version of this article (doi:10.1186/1471-2334-14-441) contains supplementary material, which is available to authorized users.

## Background

Malaria is one of the most widespread infectious diseases of our time, taking the lives of more than 650,000 people a year, most of them in sub-Saharan Africa and under the age of 5 [1]; 1300 young lives are estimated to be lost to malaria every day [2]. Malaria is holoendemic in Nigeria, accounting for 25% of infant mortality and 30% of childhood mortality [3] and transmission of malaria is geographically specific [4]. Malaria accounts for 60% of outpatient visits and 30% of hospitalizations among children under five years of age in Nigeria. In 2010, Nigeria with a population of about 150 million and reporting more deaths due to malaria than any country in the world, became the seventeenth President’s Malaria Initiative country [5]. Nigeria and Democratic Republic of Congo account for more than 40% of the total global estimated malaria deaths [6].

Information about distribution of *Anopheles* vectors and pattern of resistance to the major insecticides from Nigeria especially northern region is grossly lacking. A recent analysis of data on the distribution of *Anopheles* mosquitoes across Nigeria from 1900–2010 reported the predominance of mosquitoes from the *An. gambiae s.l.* complex (65.2%) followed by those from the *An. funestus* group (17.3%) [7]. However, most of the reports came from studies in southern Nigerian with only few studies from the north. Presently, vector control strategies involve the use of pyrethroid-based long-lasting insecticidal treated nets (LLINs) and indoor residual spray (IRS) although bendiocarb and dichlorodiphenyltrichloroethane (DDT) are also used in some areas for IRS [8]. This widespread use of a single class of insecticide could result in development of more insecticide resistance in the mosquito vectors and lead to a major public health problem given the limited availability of alternative insecticides [9].

As of May 2011 a total of 35.6 million LLINs had been distributed across 22 states of Nigeria, with a balance of 27.3 million to complete the remaining 15 states. Results from the malaria indicator survey conducted from October to December 2010, showed significant increases in LLIN ownership and use as compared to the 2008 Nigeria’s demographic health survey [5]. The average percentage for ownership of one LLIN in 2008 was 8% and increased to 42% in 2010, with rural ownership higher (45%) than urban (33%). This together with the World Bank supported IRS and insecticide treated nets (ITN) programs in several states in Nigeria [10] may add to the selective pressure on malaria vectors to develop more resistance against pyrethroids.

The two major causes of insecticide resistance in malaria vectors are alterations in the target sites and an increase in the rate of insecticide metabolism [11]. The former is characterized by knock-down resistance (*kdr*) to pyrethroids and DDT caused by mutation in the voltage-gated sodium channel (VGSC) and well characterized [12] with the L1014F*-kdr* mutation predominantly found in *An. gambiae* from West Africa and the L1014S-*kdr* mutation predominantly found in East Africa [13]. Another variant of target site mechanism of insecticide resistance in *An. gambiae* is a single amino acid substitution of glycine to serine at position 119 in the catalytic domain of the acetylcholinesterase (*AChE*) gene which confers resistance to both organophosphates and carbamates [14].

Pyrethroid knockdown resistance in *An. gambiae s.s.* from Nigeria was first reported in 2001 [15] and most of the recent studies on dominant vector species (DVS) composition and resistance profiles were carried out mostly in the south-western [16] and north-central Guinea Savannah [17] of Nigeria. Unfortunately, apart from the Garki Project carried out long time ago in 1960–1970 [18] very little has been done in the recent decades on analysis of the DVS compositions and insecticide resistance profiles of the major malaria vector from Sudan and Sahelian savannah of northern Nigeria [7]. There is thus an overwhelming need for a continued entomological survey of the malaria vector species and dissecting their resistance profile in this and other regions of Nigeria.

Here we characterised two populations of *An. gambiae s.l.* from northern Nigeria, established their species compositions, resistance profiles to insecticides, particularly pyrethroids and DDT and genotyped the main resistance markers.

## Methods

### Study site

**Figure 1:**
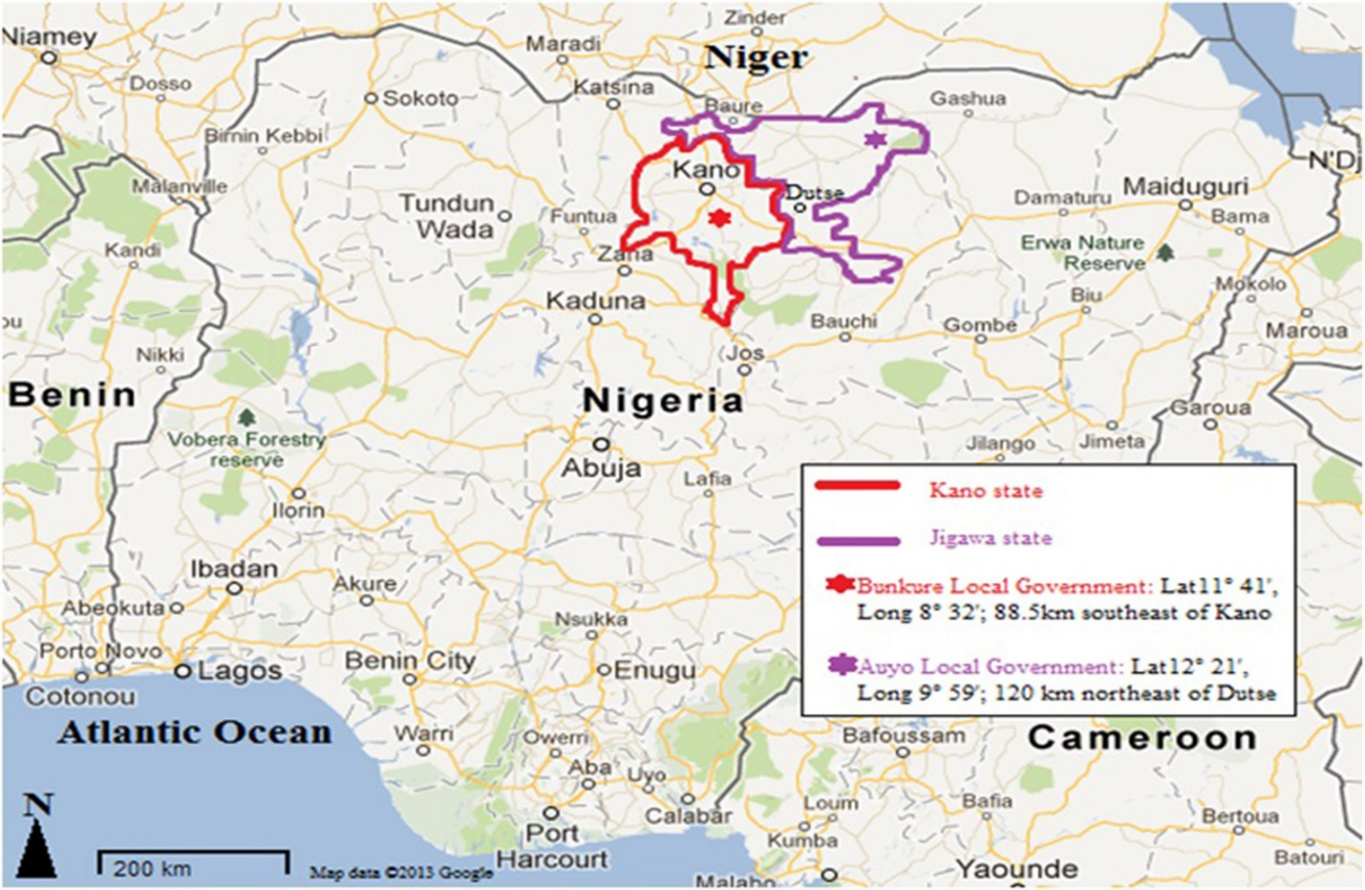
**Study Site (adopted from Google Map).**

Auyo (12°21′N, 9°59′E) located northeast of Dutse, the capital of Jigawa State (Figure 1) is a town known for its history of irrigation activities in which rice and other vegetables are produced. Jigawa state is situated within the Sudan savannah vegetation zone, but there are traces of Guinea savannah in the southern part of the state [19]. Larval collection was conducted in Auyo twice: in rainy season, June 2009 (for most bioassay tests) and during the harmattan of February 2011 (for malathion bioassay only).

Bunkure (11°42′N, 8°33′E), is a town located approximately 88.5 km east of Kano city. Kano State is located within the Sudan savannah zone of West Africa about 840 kilometers from the edge of the Sahara desert [20]. The vegetation of Kano State is semi-arid savannah sandwiched by the Sahel savannah in the north and the Guinea savannah in the south. The state has the largest irrigation projects in Nigeria, with six irrigation projects and more than twenty earth dams. Alongside the importance of these dams to the development of agriculture and provision of food comes the health implication of providing suitable breeding sites for vectors of diseases. Rice paddies in particular have been established and increase the risk of malaria by providing suitable sites for vector development. The locality of Bunkure has a large expanse of irrigable lands where rice is the major crop grown, although other crops such as green vegetables and tomatoes are grown for subsistence. Larval collections were done at the height of the rainy season in August 2010.

Both in Bunkure and Auyo farmers use petrol pumps and a myriad of pesticides to protect crops. These pesticides include organophosphates, carbamates, pyrethroids and organochlorine (including DDT and dieldrin). Reliable data on the impacts of agricultural pesticides in the wetland environments of northern Nigeria is lacking [21].

### Mosquito collection and rearing

Mosquito larvae were collected from several rice paddies within the vicinity of Bunkure town, Kano and twice in Auyo town, Jigawa using classical dipping method as described by [22]. The third and fourth instar larvae identified as belonging to the *Anopheles gambiae* complex using morphological keys [23] were transported to the insectary at Aminu Kano Teaching Hospital, Kano. The larvae were maintained under standard insectary condition (25-28°C and ~70-80% humidity, with a 12 hr day/night cycle) [24] and supplied with baby fish food daily. The adults that emerged were fed 10% sucrose solution and randomly mixed for subsequent experiments.

### Mosquito species identification

Mosquitoes were identified as belonging to the *An. gambiae s.l.* complex using the morphological keys of [23]. Genomic DNA was extracted from mosquitoes obtained from Auyo, Jigawa state and which survived exposure to lambda-cyhalothrin, using the LIVAK method [25]. The species identity of the *An. gambiae s.l.* mosquitoes and the molecular forms of all the *An. gambiae s.s.* were determined according to the SINE PCR method [26].

### Insecticide susceptibility bioassays

Insecticide susceptibility assays were carried out on 2–3 days old adult female *An. gambiae* mosquitoes following the protocol outlined by WHO [27]. Three to fifteen replicates of around 20–25 mosquitoes per tube were exposed to insecticide impregnated papers for 1 hr and then transferred to a holding tube and supplied with 10% sucrose. More replicates were used for lambda-cyhalothrin (twelve and fifteen replicates respectively for Bunkure and Auyo) with the aim of covering a large number of breeding sites in these locations. 20–25 mosquitoes were also used as a control with no exposure to any insecticide. Knockdown was recorded at intervals of 10 minutes up to an hour post-exposure to insecticides. Mortality was recorded 24 hours post-exposure to 0.05% deltamethrin, 0.05% lambda-cyhalothrin, 4% DDT and 5% malathion. Susceptible individuals are defined as individuals that did not survive a discriminating dose of the particular insecticide used. Mosquitoes from Bunkure were not tested with DDT and malathion due to these insecticide papers not being available when mosquito collection was carried out in this location.

### Genotyping of target site mutation

Presence of *kdr* and the *ace-1*^*R*^ mutations were investigated in *An. coluzzii* and *An. arabiensis* mosquitoes collected from Auyo and which survived exposure to lambda-cyhalothrin, using the pyrosequencing method as previously described [28–30]. Briefly, three sequence specific primers (Table 1) each for *kdr* and *ace-1* mutations designed using the software provided by Pyrosequencing AB (http://www.pyrosequencing.com) were used for amplification and genotyping of both mutations. Target DNA fragments for the *kdr* and *ace-1* were first PCR-amplified in a reaction containing 10 pmol each of forward and biotinylated reverse primer, 1X HotStarTaq buffer, 0.2 mM dNTPs, 1.5 mM MgCl_2_, 1U HotStarTaq (Qiagen) and 10 ng genomic DNA. The amplification was carried out using the following conditions: 1 cycle at 95°C for 5 min; 50 cycles at 94°C for 20s, 57°C for 30s and extension at 72°C for 20s; and finally 1 cycle at 72°C for 5 min. Pyrosequencing was carried out according to the manufacturer’s instructions using the PSQ 96 SNP Reagent kit (Qiagen) and the genotype determined using the SNP software (Biotage AB).

**Table 1:** ***kdr*** **and** ***AChE*** **primers**

	*Kdr (kdr-W and kdr-E)*	*ace-1* ^*R*^
Forward primer	TTGTGTTCCGTGTGCTATGC	CCTGTCCGAGGACTGTCTGT
Reverse primer	AAAAACGATCTTGGTCCATGT	ACCACGATCACGTTCTCCTC
Sequencing primer	TGTAGTGATAGGAAAT	TGTGGATCTTCGGCGG
Sequence to analyse	5′-T C/T A/T GTCGTAAG-3′	5′-C A/G GCTTCTACTCC-3′
Dispensation order for sequencing	5′-TCATcGTCGT-3′	5′-TCAGaCTCT-3′
Product size (bp)	154	165
Allele	C/T//A/T	A/G

The correlation between the L1014F *kdr* genotypes and lambda-cyhalothrin resistance phenotypes was assessed by estimating the odds ratios (OR) and the statistical significance based on the Fisher exact probability test.

## Results

### Insecticide susceptibility bioassays

**Figure 2:**
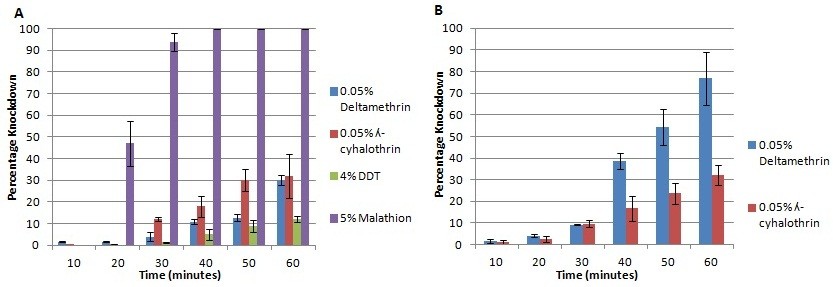
**Knockdown profile of** ***An. gambiae s.l.*** **mosquitoes: (A) Auyo (Jigawa state) and (B) Bunkure (Kano state), Nigeria.** Error bars represent variability in the data.

**Figure 3:**
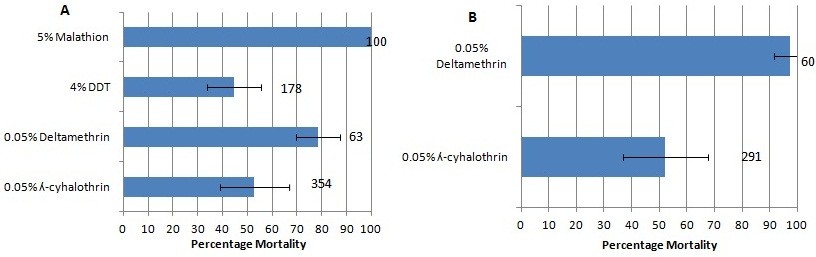
**Insecticide susceptibility/resistance status of** ***An. gambiae s.l.*** **mosquitoes: (A) Auyo (Jigawa state) and B) Bunkure (Kano state), Nigeria.** Numbers in front of the error bars represent the sample size of mosquitoes. Error bars represent variability in the data.

Insecticides bioassay conducted using the mixed F_0_ adults from Auyo, revealed resistance to DDT with only 12% knockdown after one hour exposure (Figure 2A) and mortality of 44.6% after 24 hours (Figure 3A). These mosquitoes were also resistant to lambda-cyhalothrin and deltamethrin (representative type II pyrethroids) with slow knockdown of about 30% after an hour of exposure and mortality of 52.8% and 78.4% respectively 24 hours post-exposure. However, full susceptibility was obtained with malathion (an organophosphate) with 100% knockdown within the first 40 minutes after exposure and 100% mortality after 24 hours.Mosquitoes from Bunkure were also more resistant to lambda-cyhalothrin with knockdown of only 32.6% (Figure 2B) in the first hour of exposure and mortality of 52.1% after 24 hours (Figure 3B). However, this population was found not to be resistant to deltamethrin, for 71.7% of the mosquitoes were knocked down in the first hour of exposure and 97.2% died 24 hours after exposure.

### Species and molecular forms identification

A total of one hundred and twenty one (121) females from Auyo that survived exposure to lambda-cyhalothrin and twenty six (26) dead after exposure were analysed using SINE PCR for species identification (Table 2). Approximately 77% of the mosquitoes were *An. coluzzii* (formerly M form), 22% were *An. arabiensis* and a single hybrid mosquito (<1.0%) was also found. No *An. gambiae s.s.* (formerly S form) were detected. *An. coluzzii* was predominant among the mosquitoes resistant to lambda-cyhalothrin (86.8%) while *An. arabiensis* was predominant in the susceptible mosquitoes (69.2%). Overall, 92.9% (105/113) of *An. coluzzii* were resistant to lambda-cyhalothrin whereas only 45.4% (15/33) *An. arabiensis* were resistant. Molecular analyses were not conducted with the rest of the mosquitoes used in this research for logistic reasons.

**Table 2:** **Correlation between the 1014 F allele frequency and resistance phenotypes to lambda-cyhalothrin**

Species	Phenotype	n	*L1014F alleles*		Odds ratio	P value
			TTT (R)	TTA (S)		
*An. coluzzii*	Alive	81	136	26	9.41	0.001
	Dead	7	5	9	(32.9-30.4)	
	Total	88	80.1%	19.9%		
*An. arabiensis*	Alive	13	5	21	2.85	0.42
	Dead	13	2	24		
	Total	26	13.5%	86.5%		

### Genotyping of target-site mutations

#### Knockdown resistance (kdr) mutations

A total of one hundred and fifteen (115) mosquitoes from Auyo which were exposed to lambda-cyhalothrin and identified to molecular level were successfully pyrosequenced simultaneously for both *West-kdr* (L1014F) and *East-kdr* (L1014S) mutations. This included ninety-five mosquitoes alive after exposure (81 *An. coluzzii*, 13 *An. arabiensis* and one hybrid) and twenty dead (7 *An. coluzzii* and 13 *An. arabiensis*). The pyrosequencer unambiguously scored the homozygote resistant (T/T), heterozygote resistant (A/T) and homozygote susceptible (A/A) genotypes for the L1014F-*kdr* as well as the L1014S-*kdr* genotypes.

**Figure 4:**
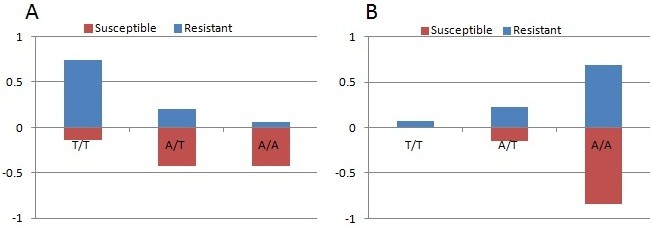
**Correlation between resistance phenotype to lambda-cyhalothrin and the L1014F** ***kdr*** **genotypes in** ***An. coluzzii*** **(A) and** ***An. arabiensis*** **(B).** The frequency of each genotype is plotted in each phenotype to indicate differences in survival between the genotypes (T/T: resistant *kdr* genotype; A/T: heterozygote; A/A: wild type susceptible).

Out of 81 genotyped *An. coluzzii* individuals alive after exposure, 74.1% (60) were homozygote (T/T) for L1014F-*kdr*, 19.7% (16) were heterozygotes, while only 6.2% (5) individuals were homozygote susceptible (A/A) (Figure 4A). Despite a low sample size of only seven *An. coluzzii* among the dead mosquitoes, a significant difference in the genotype distribution could be observed compared with the resistant mosquitoes. Consequently, a significant correlation was observed between the 1014 F allele and the resistance to lambda-cyhalothrin [OR = 9.4 (4.7-48.4); P < 0.001] (Table 2).

In contrast to *An. coluzzii*, most *An. arabiensis* resistant individuals (69.2%) were homozygote for the susceptible allele (A/A) while 23.1% (3) individuals were heterozygotes (A/T) and only 7.7% (1) homozygote for the resistant allele (T/T) (Figure 4B). Similar frequencies of the respective genotypes were observed in the dead *An. arabiensis*. Consequently, no significant correlation is observed between the 1014 F allele and the resistance to lambda-cyhalothrin in *An. arabiensis* [OR = 2.85 (0.5-16.4); P = 0.4] (Table 2).

In total, a frequency of 80.1% was detected in *An. coluzzii* for the 1014 F *kdr* resistant allele in contrast with only 13.5% in *An. arabiensis*. One *An. arabiensis* individual was found to be heterozygote for the L1014S*-kdr* mutation and homozygote susceptible (A/A) for L1014F-*kdr*. Also, the single hybrid (*An. arabiensis*/*An. gambiae*) individual genotype turned out to be heterozygote (A/T) for L1014F-*kdr* mutation.

#### Acetylcholinesterase (AChE) mutation

All the mosquitoes (100) used for bioassay with malathion were genotyped for G119S *ace-1*^*R*^ mutation. The pyrosequencer unambiguously scored all the samples with wild type G/G combination. No *ace-1*^*R*^ mutation was found in any of the mosquitoes consistent with the full susceptibility observed with malathion (Figure 3A).

## Discussion

This study has investigated the dynamics of species composition in the *An. gambiae* complex and their susceptibility status to main insecticides in northern Nigeria. Larval collection was conducted from several rice paddies in both rainy and dry season, but larger numbers of *An. coluzzii* were obtained than *An. arabiensis* in the collection during the rainy season of 2009 in Auyo, Jigawa state*.*

This supports previous observations that *An. gambiae s.s*. could be predominant in the Sudan savannah ecological zone, compared to *An. arabiensis* that was spread across Sudan, Sahel and northern Guinea savannah ecological zones [31, 32]. However, further collections are needed to fully establish the malaria vector species distribution in this region of Nigeria.

Mosquitoes from both Auyo and Bunkure localities used in this research showed a high resistance to lambda-cyhalothrin. However, only marginal resistance in mosquitoes from both sites was observed against deltamethrin. Previous studies have shown that same mosquito populations can be resistant and also susceptible to different insecticides from the same family. For example, a *kdr*-free population of *An. arabiensis* from Chad was shown to be highly resistant to permethrin but fully susceptible to deltamethrin [29]. One cytochrome P450 (*CYP6P4*) was discovered to be up-regulated in this population and shown to preferentially metabolise permethrin but not deltamethrin (S. Ibrahim, Personal Communication). Marginal resistance observed with deltamethrin indicates that *kdr* alone might not be sufficient to confer resistance to pyrethroid; it is likely that metabolic resistance is also involved. Djouaka and colleagues [33] have documented the involvement of cytochrome P450s irrespective of presence or absence of *kdr* linked with resistance in ‘M’ form of *An. gambiae* from southern Benin and south-western Nigeria. Also, Awolola and colleagues [34] have reported the presence of *kdr* in susceptible species of *An. gambiae* from south-western Nigeria and the absence of the *kdr* alleles in resistant mosquitoes of the molecular ‘M’ form and its presence in ‘S’ form [35]. Thus, metabolic resistance might play a major role in the pyrethroid resistance observed in the localities sampled, especially with respect to *An. arabiensis* which has low frequency of L1014F-*kdr* genotype.

Pyrethroid resistance observed in these *An. coluzzii* populations is similar to previously reported cases of resistance to permethrin and deltamethrin in north-central and south-western Nigeria [17, 34]. In addition, the DDT resistance observed in mosquitoes from Auyo is in keeping with previous studies elsewhere in Nigeria [16] and suggests a similar mechanism of resistance against pyrethroid and organochlorine insecticides.

Insecticide resistance was first reported in the Sudan savannah of western provinces of Sokoto, northern Nigeria back in 1958 [36] and specifically, resistance to dieldrin was first reported in *An. gambiae* from the same ecological zone in 1959 [37]. However, to our knowledge this is the first study that investigates insecticide resistance in *An. gambiae s.l.* from Sudan savannah of northern Nigeria after several decades, particularly against the pyrethroid insecticides. It is also the first of its kind to report the presence of East form of *kdr* (L1014S mutation) in *An. arabiensis* from Nigeria though the mutation has been reported in low frequency in *An. arabiensis*, and in the ‘M’ and ‘S’ molecular forms of *An. gambiae s.s.* from Burkina-Faso [38], *An. arabiensis* from Benin [39], as well as *An. arabiensis* from Sudan [40]. The low frequency of the East-*kdr* in these regions indicates that its selection is very recent or it has just recently migrated into these regions. The high frequency of West-*kdr* (L1014F) mutation observed in the *An. coluzzii* from Auyo is in contrast to observations from south-western Nigeria [34] where the *kdr* is high in frequency mainly in ‘S’ form and low in frequency in ‘M’ form of *An. gambiae*. The absence of ‘S’ form of *Anopheles gambiae* from the field collections conducted in Auyo could be explained by the observation previously made that the ‘M’ form (Mopti chromosomal form) is predominant in this type of ecological setting with a more permanent breeding site from the irrigation system [41].

The pyrethroid and DDT resistance observed in the *An. coluzzii* from this region suggests that this population is under local selective pressure. Indeed, increased usage of insecticides for agricultural purposes and/or widespread use of LLINs in the region could explain the high frequency of *kdr* mutation as recently observed in another ‘M’ form population of *An. gambiae* in a similar agricultural setting within Burkina Faso [30].

The few *An. arabiensis* genotyped exhibited low frequency of L104F-*kdr*. Unfortunately, in addition to the low number of *An. arabiensis* obtained in collection from Auyo in 2009, we don’t know the resistance level of *An. arabiensis* separately, as the bioassay results are for the two species combined. It is possible that *An. arabiensis* is as resistant as *An. coluzzii* but with less involvement of *kdr* as seen in Chad where a *kdr*-free resistant *An. arabiensis* population was reported with resistance driven mainly by P450s. Larger collection and profiling of resistance pattern of both *An. gambiae s.s.* and *An. arabiensis* from these sites separately can help to further elucidate this. There is also an overwhelming need to explore the involvement of metabolic resistance in Anopheline mosquitoes in this region using synergist assay and molecular tools.

The mosquitoes obtained from Auyo were fully susceptible to malathion and the susceptibility pattern was confirmed by the absence of the *ace-1*^*R*^ mutation in all the mosquitoes analysed. Susceptibility to malathion has already been established in several other *An. gambiae* populations across Africa such as Burkina-Faso [30] and Cameroon [42] and also in *An. funestus* another major malaria vector [43, 44]. Malathion could therefore be used as an alternative to pyrethroids and DDT in IRS campaigns in Sudan savannah of northern Nigeria.

## Conclusions

Control of malaria vector cannot be achieved without containment of resistance to insecticides and implementation of robust resistance management strategy hinges on the knowledge of the distribution and composition of the major malaria vectors and their resistance profiles. In this research we have established the temporal and spatial distribution of the two major malaria vectors *An. coluzzii* and *An. arabiensis* from Sudan savannah of northern Nigeria. We also described the pyrethroid, DDT and malathion resistance profiles in these mosquitoes as well as the high frequency of L1014F*-kdr* mutation in the *An. coluzzii* and presence of L1014S*-kdr* in low frequency in *An. arabiensis*. The *ace-1*^*R*^ mutation that confers resistance to carbamates and organophosphates is absent in the mosquitoes. This type of information could guide the Nigerian Malaria Control Programme in the choice of appropriate resistance management strategy to implement vis-à-vis different ecological zones in Nigeria.

## Authors’ original submitted files for images

Below are the links to the authors’ original submitted files for images. Authors’ original file for figure 1 Authors’ original file for figure 2 Authors’ original file for figure 3 Authors’ original file for figure 4
