# Supplementary figures and images for: High frequency of kdr L1014F is associated with pyrethroid resistance in Anopheles coluzzii in Sudan savannah of northern Nigeria
Source: BMC Infect Dis. 2014 Aug 15;14:441. doi: 10.1186/1471-2334-14-441 (PMC4147187; doi:10.1186/1471-2334-14-441)

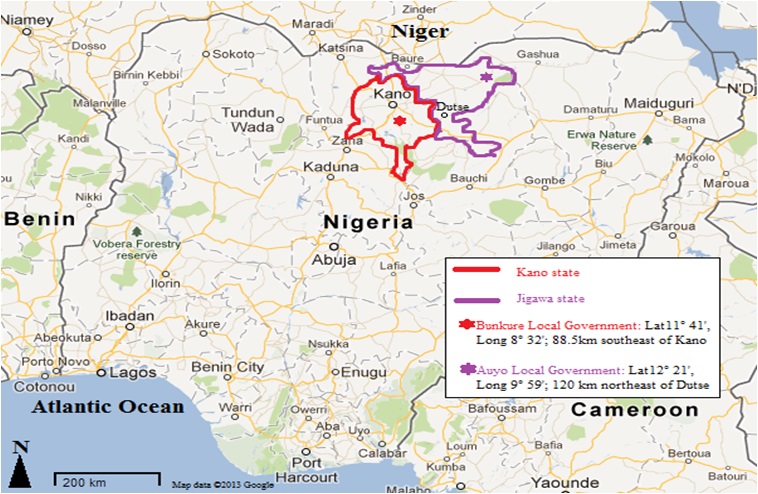

Supplement: Supplementary file 1 — Authors’ original file for figure 1 [file 12879_2014_3745_MOESM1_ESM.jpeg]

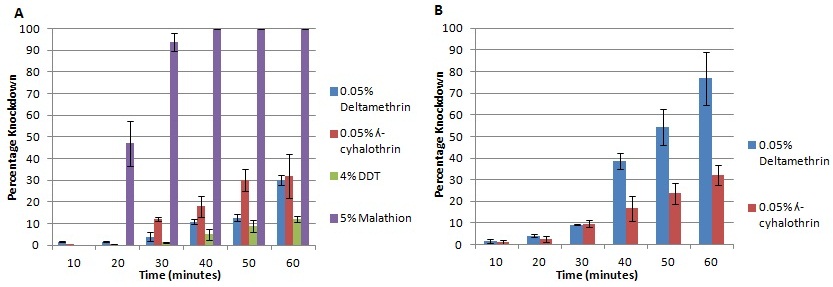

Supplement: Supplementary file 2 — Authors’ original file for figure 2 [file 12879_2014_3745_MOESM2_ESM.jpeg]

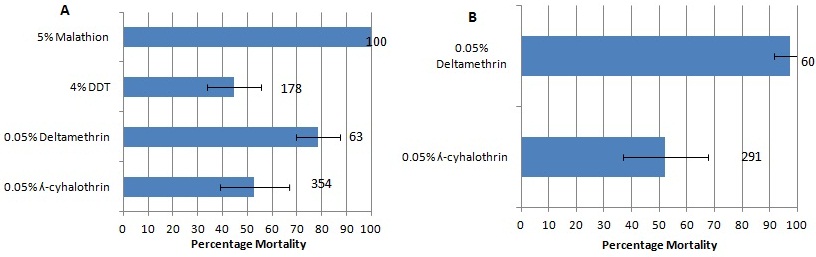

Supplement: Supplementary file 3 — Authors’ original file for figure 3 [file 12879_2014_3745_MOESM3_ESM.jpeg]

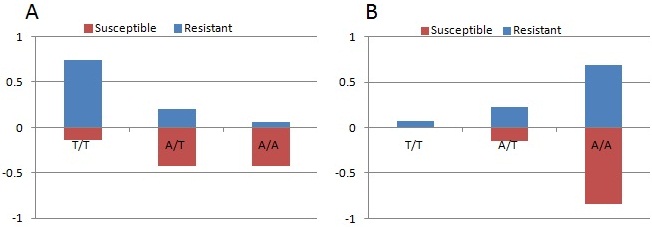

Supplement: Supplementary file 4 — Authors’ original file for figure 4 [file 12879_2014_3745_MOESM4_ESM.jpeg]
